# Induction of Human Lung Mast Cell Apoptosis by Granule Permeabilization: A Novel Approach for Targeting Mast Cells

**DOI:** 10.3389/fimmu.2017.01645

**Published:** 2017-11-27

**Authors:** Aida Paivandy, Martin Sandelin, Helena Igelström, Per Landelius, Christer Janson, Fabio R. Melo, Gunnar Pejler

**Affiliations:** ^1^Department of Medical Biochemistry and Microbiology, Uppsala University, Uppsala, Sweden; ^2^Department of Medical Sciences, Uppsala University, Uppsala, Sweden; ^3^Department of Neuroscience, Uppsala University, Uppsala, Sweden; ^4^Department of Surgical Sciences, Uppsala University, Uppsala, Sweden; ^5^Department of Anatomy, Physiology and Biochemistry, Swedish University of Agricultural Sciences, Uppsala, Sweden

**Keywords:** apoptosis, lysosomotropic agents, mast cells, granules, mefloquine

## Abstract

Mast cells are implicated as detrimental players in inflammatory lung diseases, particularly asthma. Mast cells respond to activating stimuli by releasing a wide panel of pro-inflammatory compounds that can contribute profoundly to the pathology, and there is currently an unmet need for strategies that efficiently ameliorate harmful effects of mast cells under such conditions. Here, we sought to evaluate a novel concept for targeting human lung mast cells, by assessing the possibility of selectively depleting the lung mast cells by induction of apoptosis. For this purpose, we used lysosomotropic agents, i.e., compounds that are known to permeabilize the secretory granules of mast cells, thereby releasing the contents of the granules into the cytosol. Either intact human lung tissue, purified human lung mast cells or mixed populations of human lung cells were incubated with the lysosomotropic agents mefloquine or siramesine, followed by measurement of apoptosis, reactive oxygen species (ROS) production, and release of cytokines. We show that human lung mast cells were highly susceptible to apoptosis induced by this strategy, whereas other cell populations of the lung were largely refractory. Moreover, we demonstrate that apoptosis induced by this mode is dependent on the production of ROS and that the treatment of lung tissue with lysosomotropic agents causes a decrease in the release of pathogenic cytokines. We conclude that selective apoptosis of human lung mast cells can be accomplished by administration of lysosomotropic agents, thus introducing the possibility of using such drugs as novel therapeutics in the treatment of inflammatory lung disorders such as asthma.

## Introduction

Mast cells are innate immune cells, which reside in tissues that are exposed to the external environment, such as skin, intestine, and lung. They leave bone marrow as immature myeloid cells, migrate through blood to peripheral tissues where they mature (1). Mast cells are rich in lysosome-like organelles, denoted granules, that are filled with potent preformed compounds, such as histamine, serotonin, serglycin proteoglycans, certain preformed cytokines (e.g., TNF), growth factors (e.g., VEGF), lysosomal enzymes, and proteases, the latter including tryptase, chymase and carboxypeptidase A3 (2). Upon activation, e.g., by IgE receptor (FcεRI) crosslinking, the contents of the granules are released to the exterior. Activated mast cells also produce several other compounds *de novo*, such as prostaglandins and leukotrienes, cytokines, and chemokines (3). The combined actions of all of these released compounds can cause a massive inflammatory reaction, with anaphylactic shock being a serious manifestation.

Although there is some evidence that mast cells have a beneficial function in the host defense against parasitic and bacterial infection (4, 5), it is more widely recognized that they have a major detrimental impact in allergy and asthma. Therefore, there is a large need to identify therapeutic options that can be exploited for minimizing harmful effects of mast cells. Current available options for this purpose have in common that they target only a limited fraction of all of those mediators that mast cells secrete upon activation (6, 7). However, the total impact of mast cells on any pathological setting most likely represents a sum of the effects of each of the individual pro-inflammatory compounds that are released by mast cells. Hence, effective intervention with mast cell-related effects may require the simultaneous blockade of an extensive panel of mast cell-derived products, which can be difficult to achieve (6, 7). A conceivably more efficient strategy to accomplish a full blockade of the harmful events mediated by mast cells could, therefore, be to locally eliminate harmful mast cell populations altogether [discussed in Ref. (8)]. This should preferably be achieved by inducing apoptosis rather than necrosis to avoid the inflammatory side effects that accompany necrotic cell death.

In this study, we sought to identify an efficient means of selectively eliminating human lung mast cells, with the implication of using such a strategy to block harmful mast cell-mediated activities in the context of asthma. To achieve selectivity for mast cells, it is essential that the method for inducing apoptosis is based on unique properties of mast cells, and we have previously hypothesized that a potential strategy for this purpose could be to take advantage of the unique content of secretory granules in mast cells. Since these are filled with enormous amounts of fully bioactive compounds, including proteases that potentially could trigger the apoptotic machinery, we reasoned that permeabilization of the granules and consequent release of the granular contents to the cytosol could lead to apoptosis. Moreover, since mast cells have higher content of secretory granules than has any other cell type, we reasoned that apoptosis achieved by this strategy could show selectivity for mast cells vs. other cell types. As a proof of concept for this strategy, we have previously shown that granule permeabilization, by using “lysosomotropic agents,” i.e., agents that are known to permeabilize membranes of lysosome-like organelles (9, 10), have the ability to cause mast cell apoptosis (11–13). Here, we evaluated whether this concept can be used for inducing apoptosis of human lung mast cells and, if so, whether the adopted strategy shows selectivity for mast cells vs. other cell types populating human lungs. As a major tool for the study, we used mefloquine (Lariam), a drug that has wide spread use in the treatment of malaria and also has been shown to possess lysosomotropic activity (14). In addition, we assessed the effect of siramesine, a sigma-2 agonist that has been found to have lysosomotropic activity (15, 16). We show, in accordance with our hypothesis, that lysosomotropic agents efficiently and selectively induce apoptosis of human lung mast cells. Based on these findings, we propose that lysosomotropic agents potentially could be explored as therapeutics for lung diseases where mast cells contribute, e.g., asthma.

## Materials and Methods

### Reagents

Mefloquine and N-acetylcysteine (NAC) were purchased from Sigma-Aldrich (St. Louis, MO, USA). A stock solution of 50 mM mefloquine was prepared in dimethyl sulfoxide. This solution was further diluted using phosphate-buffered saline (PBS) to prepare the final working solution of mefloquine. Siramesine hydrochloride, a generous gift by Lundbeck A/S (Copenhagen, Denmark), was prepared as a stock solution of 1 mM in 10% HPBCD ([2-hydroxypropyl]-beta-cyclodextrin) (Sigma-Aldrich) and subsequently diluted in PBS for the further use in experiments.

### Cell Culture

Bone marrow-derived mast cells (BMMCs) from C57BL/6 mice were generated as described (17). Cells that were at least four weeks old were used for experiments. Primary human lung smooth muscle cells (ATCC, Manassas, VA, USA) were cultured according to the manufacturer’s instructions.

### Lung Tissue Samples and Ethics

Tumor-free parts of lung tissue were obtained from patients undergoing surgical lung resection due to lung cancer. The tissues were immersed in DMEM containing GlutaMAX™ supplement and placed on ice immediately after surgical excision. Written informed consent was obtained from all patients, and the use of the patient samples for this study was approved by the Uppsala Regional Ethical Review Board (Dnr 2013/223).

### Immunohistochemistry and *In Situ* Apoptosis Assessment

Lung specimens (ranging from 1 to 4 g) were cut into equal-sized pieces and placed in 6-well plates containing DMEM (Dulbecco’s Modified Eagle Medium) supplemented with 10% heat-inactivated fetal bovine serum (FBS), 2 mM l-glutamine, 100 U/mL penicillin, and 100 µg/mL streptomycin. The samples were incubated with mefloquine, siramesine, or vehicle (PBS) for 20–24 h in a humidified 37°C incubator with 5% CO_2_. Treated tissues were fixed in 4% formalin, embedded in paraffin and, 5 µm sections were cut. Sections were deparaffinized and boiled in a pressure cooker (Reveal Decloaker, Biocare Medical, Concorde, CA, USA). Background sniper (Biocare Medical) was used to block non-specific background staining. For mast cell detection, the sections were incubated with a monoclonal tryptase antibody (MAB1222, Millipore, Chemicon International Inc., Temecula, CA, USA) at 1/2,000 dilution overnight, followed by visualization by applying the MACH 3 Mouse AP-Polymer Detection kit and Vulcan Fast Red Chromogen Kit 2 (Biocare Medical). The sections were counterstained with Mayer’s hematoxylin (Histolab, Gothenburg, Sweden). Incubation with mouse IgG was used as negative control. For assessment of mast cell apoptosis *in situ*, TUNEL-tryptase double staining was carried out using the ApopTag plus Peroxidase *in Situ* Apoptosis Detection Kit (Millipore, Billerica, MA, USA) and monoclonal tryptase antibody as described above.

### Extraction and Preparation of Lung Cells

Human lung tissues were digested using the Human Tumor Dissociation Kit and the gentleMACS Octo Dissociator (all from Miltenyi Biotec, Bergisch Gladbach, Germany) according to the manufacturer’s instructions. Tissue residues were removed using a 70-µm cell strainer followed by centrifugation at 300 × *g* for 8 min at 4°C. Red blood cells were lysed using Red Blood Cell Lysis Solution (Miltenyi Biotec). The number of viable cells was determined by trypan blue exclusion using a hemocytometer. Extracted lung cells were resuspended in DMEM containing GlutaMAX™ supplement (Product No. 10564–011, Life Technologies, Carlsbad, CA, USA), 10% heat-inactivated FBS, 100 U/mL penicillin, 100 µg/mL streptomycin and 1 × MEM non-essential amino acids and were subsequently seeded in 24-well plates at a concentration of 0.5 × 10^6^ cells/well. The cells were then incubated with mefloquine or PBS in a humidified 37°C incubator with 5% CO_2_ and the cytotoxicity of mefloquine was examined by flow cytometry. For experiments shown in Figures 2C,D, after removal of red blood cells, c-kit^+^ lung cells were separated using anti c-kit-coated magnetic beads (Miltenyi Biotec) and a MACS column. Purified c-kit^+^ lung cells were seeded and treated with mefloquine or PBS as described above.

### Monoclonal Antibodies, Flow Cytometry, and Apoptosis Evaluation

Mixed populations of human lung cells (obtained after mechanical and enzymatic digestion of lung specimens) were stained with fluorochrome-conjugated monoclonal antibodies against the following surface markers: CD4 (RPA-T4), CD8 (RPA-T8), CD14 (M5E2), CD19 (HIB19), CD45 (HI30), c-kit (104D2), FcεRI (AER-37), and CD326 (EBA-1). The antibodies were from BD Biosciences (Franklin Lakes, NJ, USA) or BioLegend (San Diego, CA, USA). To evaluate apoptosis/necrosis, mixed populations of human lung cells, human lung c-kit^+^ cells or primary human lung smooth muscle cells were treated with PBS or mefloquine and then stained with Annexin V (BD Biosciences, Franklin Lakes, NJ, USA) and DRAQ7™ (Biostatus Ltd., Shepshed, UK). To determine the effect of NAC on cell death, extracted lung cells were pre-incubated with or without NAC (8 mmol/L) for 2 h and subsequently treated with mefloquine or PBS for 24 h. The cells were stained for mast cell surface markers and Annexin V/DRAQ7 and analyzed on a LSR II or LSR Fortessa flow cytometer (BD Biosciences), and data analysis was performed using the FlowJo software (TreeStar Inc., Ashland, OR, USA). In all flow cytometry analyses, doublet cells were excluded.

### Measurement of Reactive Oxygen Species (ROS)

Extracted lung cells were treated with mefloquine or PBS for 1 h. The cells were washed with PBS and incubated for 30 min with 5 µM CM-H_2_DCFDA (Life Technologies, Carlsbad, CA, USA) in PBS at 37°C in dark. After washing, the cells were stained with monoclonal antibody against human c-kit and subsequently analyzed for ROS production by flow cytometry.

### Cell Visualization

Human lung c-kit^+^ (sorted by magnetic-activated cell sorting) and/or c-kit^+^ FcεRI^+^ cells (sorted by fluorescence-activated cell sorting) were cytospun onto glass slides (Shandon Cytospin 2) and allowed to dry overnight. The cells were stained with toluidine blue (Sigma-Aldrich) using a standard protocol. Tryptase activity was detected by incubation of cytospin slides with a solution of 10 mM Z-Gly-Pro-Arg 4-methoxy-2-naphtylamine in 0.5 M Tris–HCl (pH 7.5) and 5 mg/mL Fast Garnet GBC sulfate salt (all from Sigma-Aldrich).

### Microscopic Image Analysis

All images were captured using a Nikon Eclipse 90i microscope (Nikon, Melville, NY, USA) and NIS Elements AR 3.2 64-bit software (Nikon) at original magnifications of 20× or 40×.

### ELISA

Lung tissue specimens were prepared and incubated with either mefloquine, siramesine, or PBS as described before (see Immunohistochemistry and *In Situ* Apoptosis Assessment). After 6 h, supernatants were collected for measurement of VEGF and IL-6 by ELISA. The ELISA kits for human VEGF and IL-6 were both from Sigma-Aldrich. The ELISA measurements were carried out according to the instructions provided by the manufacturer.

### Statistical Analysis

In figures representing two groups, statistical differences between groups were assessed using unpaired, two-tailed Student’s *t*-test. In figures with more than two groups, one-way ANOVA with *post hoc* Tukey’s multiple comparison test was used to determine statistical significance between groups. All graphs were prepared and statistics calculated using GraphPad Prism 7.0 (GraphPad software Inc., San Diego, CA, USA). A *P*-value of less than 0.05 was considered significant.

## Results

### Mefloquine and Siramesine Reduce Mast Cell Numbers in Human Lung Tissue *In Situ*

**Figure 1:**
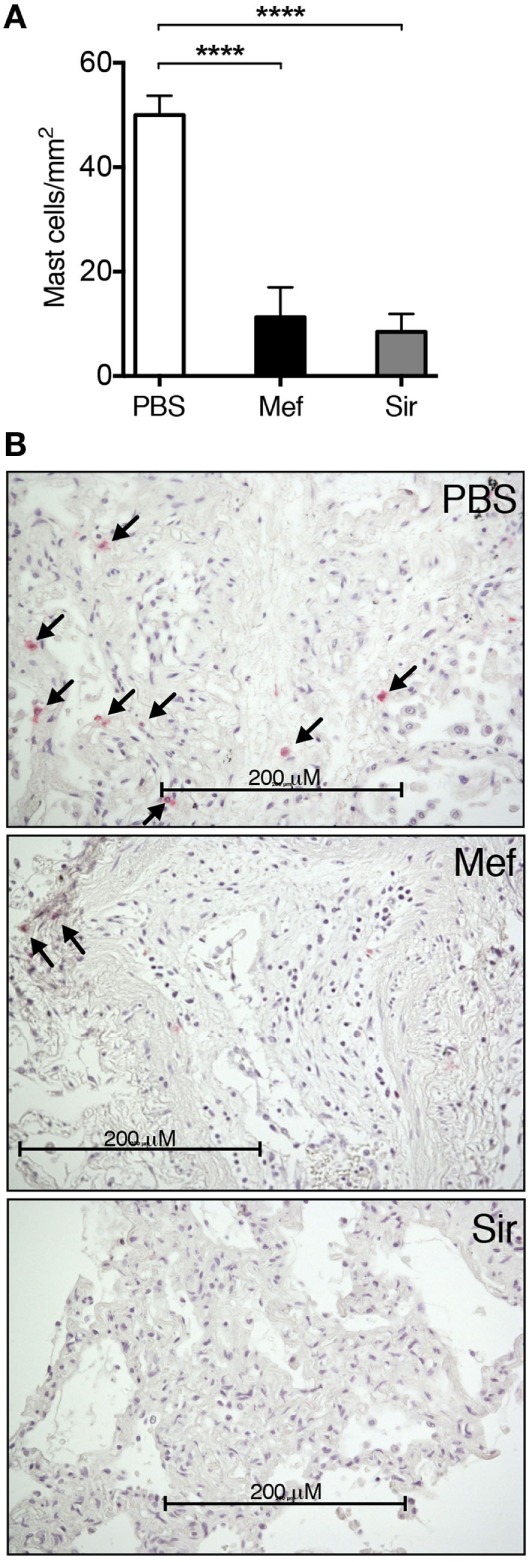
Mefloquine and siramesine reduce the number of mast cells in human lung tissues. Human lung specimens were incubated in media containing 20 µM of mefloquine, siramesine, or phosphate-buffered saline (PBS; as vehicle) for 24 h. Cross sections of the lung specimens were then prepared and immunostained for tryptase followed by nuclear counterstaining with Mayer’s hematoxylin to evaluate the number of lung mast cells. **(A)** Quantification of the number of lung tryptase^+^ mast cells (*n* = 5–10; representative of two independent experiments/two donors). The graphs show mean ± SEM (****P < 0.0001). **(B)** Representative images of lung sections showing the reduction in the number of tryptase^+^ mast cells (arrows) in the presence of mefloquine or siramesine.

To assess the effect of lysosomotropic agents, mefloquine and siramesine, on human lung mast cells, specimens were obtained from the non-tumor parts of lung tissue dissected during lung cancer surgery. These tissue specimens were incubated in culture medium containing either mefloquine (20 µM), siramesine (20 µM) or PBS as control. After 24 h of incubation, the lung specimens were sectioned and subsequently stained for tryptase to identify mast cells; tryptase is a major component of the secretory granules of human lung mast cells of both MC_T_ and MC_TC_ subtypes (18). As depicted in Figure 1, there was a profound reduction in the number of tryptase^+^ mast cells in the lung tissues after treatment with either mefloquine or siramesine in comparison with vehicle-treated tissues. Of note, there was no sign of tissue destruction caused by mefloquine or siramesine treatment. Hence, these results show that both mefloquine and siramesine efficiently reduce the number of mast cells in human lung tissue *in situ*. Of these compounds, mefloquine is an approved drug (for malaria treatment) whereas siramesine is reported to be safe in humans (19) but not approved for clinical use. In the continuation of this study, we therefore focused primarily on mefloquine, the rationale being that this approved compound has a higher potential (than siramesine) of becoming rapidly adapted to clinical usage.

### Mefloquine Induces Apoptotic Cell Death in Human Lung Mast Cells

**Figure 2:**
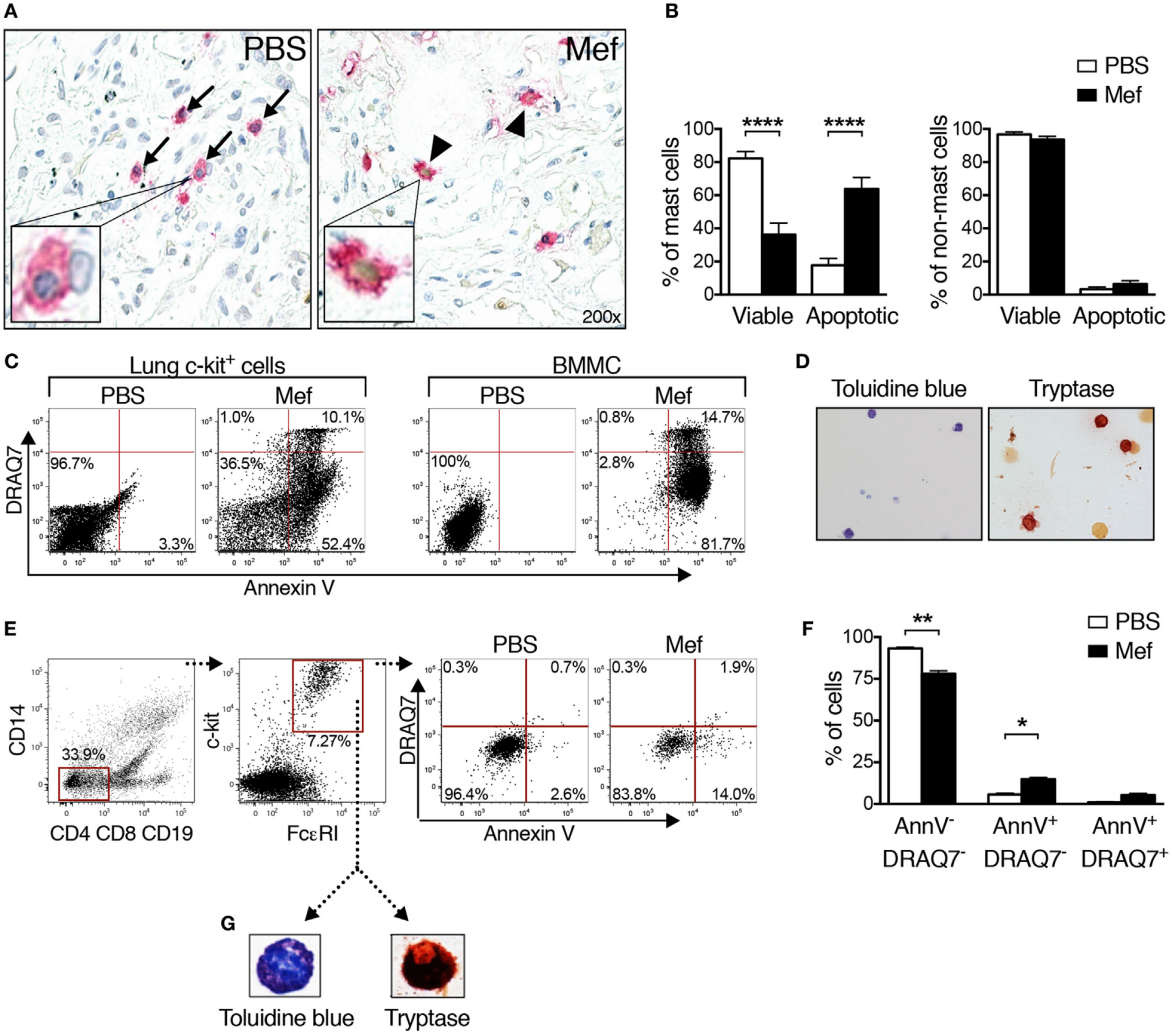
Mefloquine induces apoptotic cell death in human lung mast cells. Human lung specimens were incubated with mefloquine (Mef; 20 µM) or phosphate-buffered saline (PBS) for 20 h. TUNEL-tryptase double staining was performed on cross sections of the lung biopsies followed by nuclear counterstaining with Mayer’s hematoxylin. **(A)** Representative images of lung sections showing the reduction in the number of viable mast cells (TUNEL^−^/tryptase^+^, blue nucleus with pink cytoplasm, arrows) and increase in the number of apoptotic mast cells (TUNEL^+^/tryptase^+^, brown nucleus with pink cytoplasm, arrowheads). The inserts in panel A show enlarged images of viable (left) or apoptotic (right) mast cells. **(B)** Percentage of viable (TUNEL^−^/tryptase^+^) and apoptotic mast cells (TUNEL^+^/tryptase^+^) (left panel, *n* = 20; representative of two independent experiments/two donors) and percentage of viable (TUNEL^−^/tryptase^−^) and apoptotic non-mast cells (TUNEL^+^/tryptase^−^) (right panel, *n* = 4; representative of two independent experiments/two donors) in the lung tissue after treatment with PBS or mefloquine. Results are shown as mean ± SEM (****P < 0.0001). **(C)** Mixed human lung cells were prepared by mechanical and enzymatic treatment of lung specimens and c-kit^+^ cells were enriched by magnetic-activated cell sorting. Human lung c-kit^+^ cells or murine cultured mast cells [bone marrow-derived mast cells (BMMCs); used as controls] were treated with mefloquine (20 µM) or PBS for 24 h. Cells were stained with Annexin V and DRAQ7 and cell death was assessed by flow cytometry. Representative dot plots in panel **(C)** display the Annexin V/DRAQ7 staining of human lung c-kit^+^ cells (left) and BMMCs (right) after treatment with PBS or mefloquine. The results in panel **(C)** are representative of two independent experiments (two donors and two BMMC cultures). **(D)** Representative images of human lung c-kit^+^ cells stained with toluidine blue (left) and for tryptase activity (right panel). **(E)** Mixed human lung cells were prepared and incubated with PBS or mefloquine (10 µM) followed by assessment of cell death by flow cytometry. Representative dot plots in panel **(E)** demonstrate the gating strategy used to identify lung mast cells (Lin^−^ c-kit^+^ FcεRI^+^) and Annexin V/DRAQ7 staining after treatment with PBS or mefloquine. **(F)** Quantification of viable (Annexin V^−^/DRAQ7^−^), apoptotic (Annexin V^+^/DRAQ7^−^), and necrotic (Annexin V^+^/DRAQ7^+^) Lin^−^ c-kit^+^ FcεRI^+^ mast cells (*n* = 4; representative of four independent experiments/four donors). Data are presented as mean ± SEM (*P < 0.05, **P < 0.01). **(G)** Lin^−^ c-kit^+^ FcεRI^+^ cells were sorted and collected onto cytospin slides and subsequently stained with toluidine blue or for tryptase activity.

Next, we sought to determine the mechanism underlying the reduction of mast cell numbers in lung tissues incubated with mefloquine. For this purpose, we first double stained mefloquine-treated and control lung tissue sections for tryptase and with TUNEL, the latter is an assay that detects apoptotic cells. As seen in Figure 2A (left panel), mast cells (tryptase^+^) in the control sections were generally TUNEL-negative, i.e., have a non-stained (blue) nucleus. By contrast, a large fraction of the mast cells in mefloquine-treated lung tissues were TUNEL-positive, i.e., their nuclei showed brown staining that is characteristic of TUNEL-positivity (Figure 2A; right panel). Quantification of these data showed that the treatment with mefloquine caused a substantial and significant decrease in the number of viable mast cells (tryptase^+^/TUNEL^−^) accompanied by a significant increase in the number of apoptotic mast cells (tryptase^+^/TUNEL^+^) (Figure 2B; left panel). By contrast, non-mast cells were minimally affected by the mefloquine treatment (Figure 2B; right panel), suggesting that mefloquine shows selectivity for mast cells.

The effect of mefloquine on human lung mast cells was further evaluated by a flow cytometry-based approach that enables a quantitative distinction between apoptotic and necrotic cell death. For this, lung specimens were mechanically and enzymatically digested to extract the lung cells. Among cells of human lung, mast cells express high levels of c-kit on their surface (20, 21). Thus, to purify mast cells, c-kit^+^ cells were selected from extracted lung single cell suspensions, using magnetic cell sorting enrichment. The c-kit^+^ cells were then treated with mefloquine or PBS followed by flow cytometry-based analysis of the cell death mechanism using Annexin V and DRAQ7 staining. As controls, mouse BMMCs were also assessed. In agreement with our previous results (12), treatment of BMMCs with mefloquine mainly resulted in appearance of apoptotic cells (Annexin V^+^/DRAQ7^−^), whereas a minor fraction of the cells showed signs of necrotic cell death (Annexin V^+^/DRAQ7^+^) (Figure 2C; right panel). Similarly, the majority of the mefloquine-treated human lung c-kit^+^ cells were found to be apoptotic rather than necrotic (Figure 2C; left panel), reinforcing that mefloquine predominantly triggers apoptotic cell death of human lung mast cells.

To confirm that the selection based on c-kit expression results in purification of mast cells, the purified c-kit^+^ cells were stained with toluidine blue staining and for tryptase activity. Indeed, a substantial fraction of the c-kit^+^ lung cells stained positively with toluidine blue and for tryptase activity, indicating that they represented a population of enriched mature mast cells (Figure 2D).

### Mefloquine Has No Adverse Effect on Other Immune or Structural Cells of the Lung

Next, we implemented an experimental strategy where the relative extent of cell death of the individual cell types present in the human lung can be studied. To this end, mixed cell suspensions were prepared by mechanical and enzymatic digestion of the human lung specimens and were treated with mefloquine or PBS. Individual cell types were then identified by flow cytometry and their extent of cell death was evaluated by Annexin V/DRAQ7 staining. Human lung mast cells were thereby identified as being negative for expression of lineage markers CD4, CD8, CD19, and CD14 (Lin^−^) and positive for c-kit and FcεRI (Figure 2E). Monocytes/macrophages were identified as CD14^+^ and T/B lymphocytes as CD4^+^ CD8^+^ CD19^+^ cells, respectively. Epithelial cells were identified as (CD45^−^ CD326^+^). No cell surface markers specific for the airway smooth muscle cell population are to date available. To study the effect of mefloquine on this population, we therefore used purified primary human lung smooth muscle cells.

**Figure 3:**
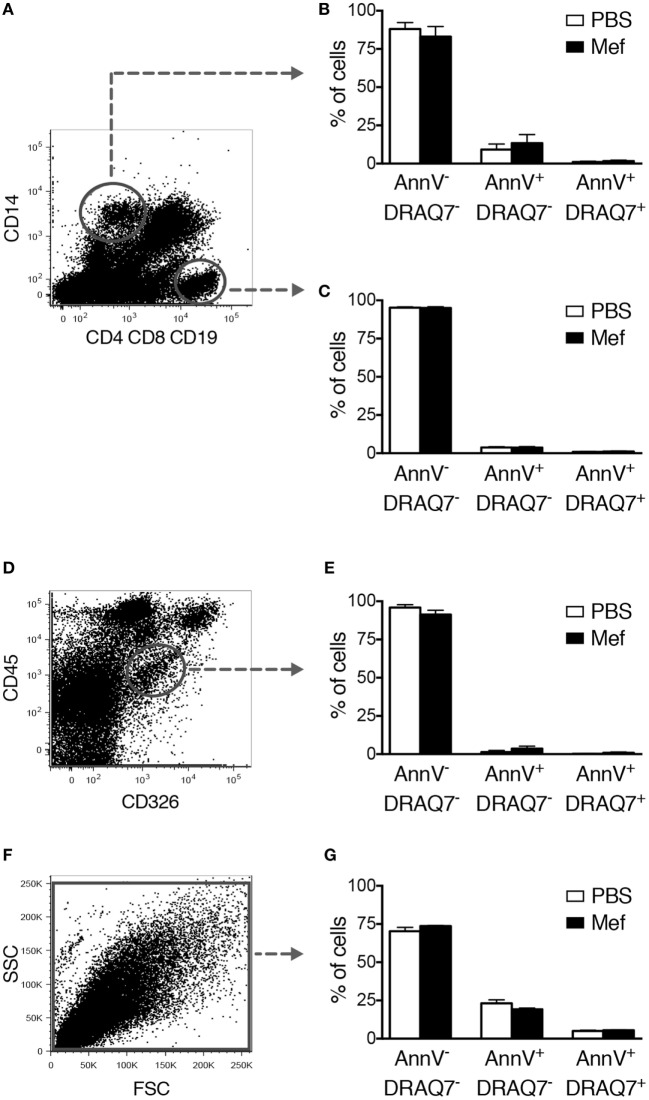
Mefloquine has no adverse effect on non-mast cell populations of the lung. Mixed human lung cells were prepared by mechanical and enzymatic treatment of lung specimens and were incubated with mefloquine (10 µM) or phosphate-buffered saline (PBS) for 24 h and then stained with antibodies recognizing the indicated surface markers. Subsequently, cell death was evaluated by flow cytometry. **(A)** Representative dot plots showing gated human lung CD14^+^ cells (monocytes/macrophages) and CD4^+^ CD8^+^ CD19^+^ cells (T/B lymphocytes). **(B,C)** Quantification of viable (Annexin V^−^/DRAQ7^−^), apoptotic (Annexin V^+^/DRAQ7^−^), and necrotic (Annexin V^+^/DRAQ7^+^) human lung CD14^+^ **(B)** or CD4^+^ CD8^+^ CD19^+^ **(C)** cells (*n* = 4; representative of two independent experiments/two donors). **(D,E)** Human lung epithelial cells (CD45^−^ CD326^+^) were gated **(D)** and the percentage of viable, apoptotic, and necrotic cells were determined by flow cytometry **(E)** (*n* = 3; representative of two independent experiments/two donors). **(F,G)** Primary human lung smooth muscle cells were incubated with mefloquine or PBS as described above and cell death was evaluated by flow cytometry (*n* = 3; representative of two independent experiments). All data are presented as mean ± SEM.

Similar to the purified c-kit^+^ lung cells, Lin^−^ c-kit^+^ FcεRI^+^ mast cells present in the mixed lung cell suspensions underwent apoptosis after treatment with mefloquine (Figures 2E,F). To verify that this strategy identifies mast cells, sorted human lung Lin^−^ c-kit^+^ FcεRI^+^ cells were stained with toluidine blue^+^ and for tryptase activity. As shown in Figure 2G, the sorted Lin^−^ c-kit^+^ FcεRI^+^ cells stained positively both with toluidine blue and for tryptase activity, confirming that the gating strategy employed identifies mast cells. These data further support that human lung mast cells are sensitive to mefloquine and undergo apoptosis in response to this lysosomotropic agent. By contrast, mefloquine at concentrations that were cytotoxic for mast cells in this setting had no significant cytotoxic activity toward CD14^+^ or CD4^+^ CD8^+^ CD19^+^ cells, i.e., monocytes/macrophages or T/B lymphocytes, respectively (Figures 3A–C). Moreover, mefloquine treatment did not induce cell death in epithelial cells (CD45^−^ CD326^+^) (Figures 3D,E) or in primary human lung smooth muscle cells (Figures 3F,G), both of which representing major structural cells of the lung. Taken together, these data show that mefloquine has limited adverse effects on lung cell populations other than mast cells, i.e., mefloquine shows selectivity for mast cells.

### Mefloquine-Induced Oxidative Stress Is the Major Cause of Mast Cell Apoptosis

**Figure 4:**
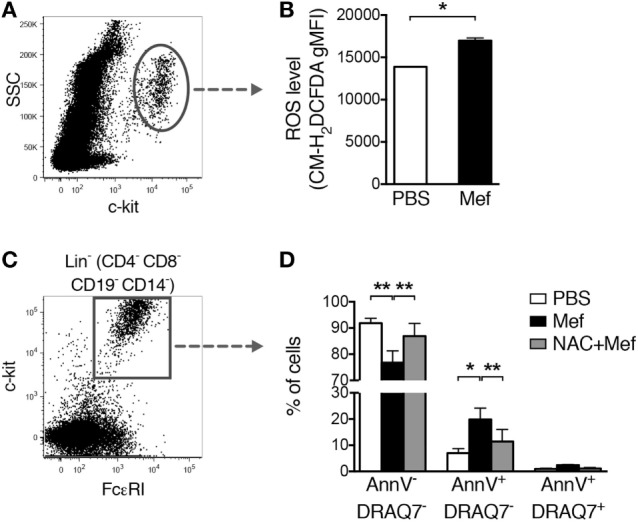
Oxidative stress induced by mefloquine is a major cause of mast cell apoptosis. **(A,B)** Mixed human lung cells were prepared by mechanical and enzymatic treatment of lung specimens and treated with phosphate-buffered saline (PBS) or mefloquine (20 µM) for 1 h. Next, c-kit^+^ cells were gated **(A)** and the reactive oxygen species (ROS) levels were quantified using the fluorescent ROS probe CM-H_2_DCFDA **(B)**. The bars in panel **(B)** represent mean ± SEM of the geometric mean fluorescence intensity (gMFI) for the CM-H_2_DCFDA (*P < 0.05) (*n* = 2; representative of two independent experiments/two donors). **(C,D)** Mixed human lung cells were pre-incubated with or without N-acetylcysteine (NAC) (8 mmol/L) for 2 h, followed by treatment with mefloquine (20 µM) or PBS for 24 h. Lin^−^ c-kit^+^ FcεRI^+^ mast cells were gated **(C)** and the percentage of viable (Annexin V^−^/DRAQ7^−^), apoptotic (Annexin V^+^/DRAQ7^−^) and necrotic (Annexin V^+^/DRAQ7^+^) mast cells were determined **(D)** (*n* = 2; representative of three independent experiments/three donors). Data are presented as mean ± SEM (*P < 0.05, **P < 0.01).

Next, we addressed the mechanism of cell death in response to lysosomotropic agents such as mefloquine. In a previous study, we showed that treatment of murine cultured mast cells (BMMCs) with a lysosomotropic agent caused the production of ROS and that the production of ROS had a central role in the induction of cell death (12). To investigate the impact of ROS on cell death in human lung mast cells exposed to lysosomotropic agents, mixed human lung cells were treated with mefloquine followed by flow cytometric analysis of ROS production using a fluorescent probe, CM-H_2_DCFDA. This analysis showed that treatment of the human lung cells with mefloquine induced significant ROS production in lung mast cells (c-kit^+^ cells) (Figures 4A,B). Moreover, treatment of the cells with NAC, a ROS scavenger, blocked apoptotic cell death induced by mefloquine (Figures 4C,D). Hence, these data suggest that ROS production plays an essential role in the pathway leading to mast cell death in response to lysosomotropic agents.

### Lysosomotropic Agents Reduce VEGF and IL-6 Levels in Human Lung Tissue

**Figure 5:**
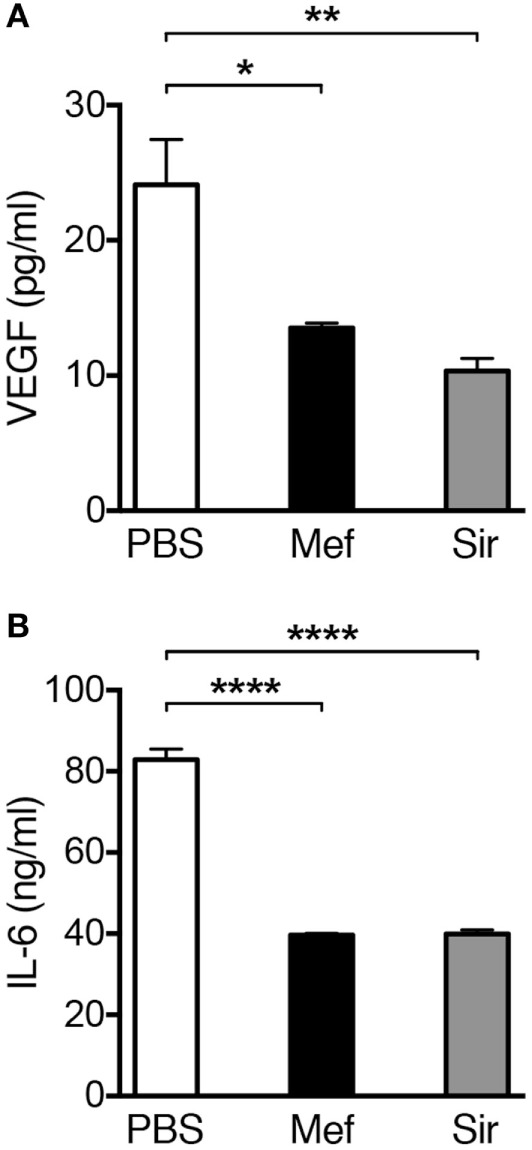
Treatment of human lung biopsies with mefloquine and/or siramesine reduces the levels of VEGF and IL-6. Human lung biopsies were incubated with 20 µM of mefloquine or siramesine or phosphate-buffered saline (PBS) for 6 h. Levels of **(A)** human VEGF and **(B)** IL-6 were measured in the supernatants by ELISA (*n* = 3; representative of two independent experiments/two donors). Results are presented as mean ± SEM (*P < 0.05, **P < 0.01, ****P < 0.0001).

Mast cells are known to be the source of multiple cytokines and growth factors with pathogenic roles in the context of inflammatory lung diseases (3). For example, mast cells are major producers of VEGF and IL-6, both of which having the potential to contribute significantly to the pathology of inflammatory disorders, e.g., asthma (22–24). In order to investigate if treatment with lysosomotropic drugs can suppress the levels of these compounds in lung tissue, we used ELISA. As shown in Figure 5A, incubation of lung tissue specimens with mefloquine or siramesine resulted in decreased levels of VEGF in supernatants recovered from lung tissue specimens. The treatment with either mefloquine or siramesine also caused a significant reduction of IL-6 levels (Figure 5B). Hence, exposure of lung tissue to lysosomotropic agents has the potential to suppress the levels of mast cell-expressed pathogenic cytokines.

## Discussion

Mast cells are currently emerging as major detrimental players in inflammatory lung diseases, in particular allergic asthma. There is now vast clinical documentation supporting that mast cells contribute profoundly to the pathology of asthma (25–28). Moreover, asthmatic patients have increased numbers of lung mast cells, especially in locations such as the airway smooth muscle layer, lung epithelium, and alveolar parenchyma (25, 29, 30). Of note, the abnormal accumulation of mast cells in these lung compartments has been associated with enhanced asthma symptoms (25, 30–32). In addition to allergic asthma, mast cells have also been implicated in other types of lung pathologies, such as idiopathic pulmonary fibrosis and COPD (33, 34). Interestingly, a role for mast cells in asthma is also supported by a number of studies conducted on mice. In mouse models of allergic asthma, elevated numbers of airway mast cells were found (35) and mast cells have been shown to contribute in a major way to several symptoms associated with experimentally induced allergic airway inflammation (35). Furthermore, studies on mice have suggested that mast cells and their products can contribute in models of lung fibrosis (36) and COPD (37, 38).

Taken together, there is, therefore, a large interest in developing novel therapeutic regimens for these types of diseases, in which lung mast cells are targeted. Currently, available therapies for this purpose include the usage of antihistamines, leukotriene receptor antagonists, mast cell stabilizers, and anti-IgE therapy (6, 7). However, neither of these treatment options is capable of suppressing the full panel of events mediated by mast cells, i.e., only a fraction of all of the mediators that are secreted by mast cells is blocked by these treatment options. There is, therefore, a need for more efficient strategies to prevent harmful mast cell actions, and we have reasoned that this could potentially be achieved by selectively and locally inducing mast cell apoptosis. If successful, such a strategy would have the capacity to abrogate the production and secretion of the full panel of pathogenic compounds in tissue mast cells, in this way efficiently interfering with the detrimental impact of mast cells on inflammatory conditions. Here, we show that efficient and selective apoptosis of mast cells can be induced by exposure of either intact lung tissue specimens, purified lung mast cells or lung mast cells present in mixed cell suspension to lysosomotropic agents. Furthermore, we demonstrate that treatment of mixed human lung cells with mefloquine triggered an oxidative stress in lung mast cells, which was evident by enhanced ROS production. Here, the mefloquine-induced ROS production was found to be the major cause of mast cell apoptosis because pre-incubation of mixed human lung cells with NAC, a ROS scavenger, inhibited mast cell apoptosis in response to mefloquine. These results are in agreement with our previous findings, which showed that increased ROS production is responsible for murine BMMC death upon exposure to mefloquine (12).

Mast cells can contribute to the pathology of inflammatory lung diseases, for example, through production and secretion of several growth factors and cytokines. VEGF and IL-6 are among these compounds that have been linked to asthma pathology (22–24). VEGF is a growth factor with potent angiogenic activity that is elevated in asthma and was shown to promote vascular remodeling, permeability, and chemotaxis of inflammatory cells (39–42). Moreover, increased levels of IL-6 in serum, bronchoalveolar lavage fluid, and sputum have been found in asthmatics (43–46). Interestingly, we found that treatment of human lung tissues with lysosomotropic agents (mefloquine and siramesine) suppressed the concentrations of VEGF and IL-6.

As an extension of the findings presented here, we may envision the usage of lysosomotropic agents in the treatment of inflammatory conditions of the lung where mast cells are known to contribute to the pathology. In particular, we foresee that this type of compounds may be useful in asthma therapy, as an inhalation treatment. Potentially, such agents could be useful both during exacerbations to counteract harmful effector functions of mast cells, but also between asthma attacks to suppress the harmful mast cell populations residing in the airway epithelium (29) and smooth muscle layer (25). In this context, it is important to note that one of the lysosomotropic compounds used in this study, i.e., mefloquine, is an approved drug and that the second lysosomotropic agent used, siramesine, has been shown to be safe for use in humans (19). It would, therefore, be feasible to adapt these compounds to clinical usage in a relatively near future. In support of a potential usefulness of mefloquine as an anti-mast cell agent, previous experiments performed in mice have suggested that mefloquine selectively targets mast cells *in vivo* (12).

## Ethics Statement

Uppsala Regional Ethical Review Board (Dnr 2013/223).

## Author Contributions

AP, FM, and GP designed the study; AP performed and analyzed the experiments; MS, CJ, HI, and PL coordinated the collection of the lung samples; and AP and GP interpreted the data and wrote the manuscript.

## Conflict of Interest Statement

The authors declare that the research was conducted in the absence of any commercial or financial relationships that could be construed as a potential conflict of interest.

## Abbreviations

NAC, N-acetylcysteine; BMMC, bone marrow-derived mast cells; ROS, reactive oxygen species.

## References

[1] Gurish MF Austen KF. Developmental origin and functional specialization of mast cell subsets. Immunity (2012) 37:25–33. 10.1016/j.immuni.2012.07.003 22840841

[2] Wernersson S Pejler G Mast cell secretory granules: armed for battle. Nat Rev Immunol (2014) 14:478–94. 10.1038/nri3690 24903914

[3] Galli SJ Nakae S Tsai M. Mast cells in the development of adaptive immune responses. Nat Immunol (2005) 6:135–42. 10.1038/ni1158 15662442

[4] Marshall JS. Mast-cell responses to pathogens. Nat Rev Immunol (2004) 4:787–99. 10.1038/nri1460 15459670

[5] Johnzon CF Rönnberg E Pejler G. The role of mast cells in bacterial infection. Am J Pathol (2016) 186:4–14. 10.1016/j.ajpath.2015.06.024 26477818

[6] Reber LL Frossard N. Targeting mast cells in inflammatory diseases. Pharmacol Ther (2014) 142:416–35. 10.1016/j.pharmthera.2014.01.004 24486828

[7] Harvima IT Levi-Schaffer F Draber P Friedman S Polakovicova I Gibbs BF Molecular targets on mast cells and basophils for novel therapies. J Allergy Clin Immunol (2014) 134:530–44. 10.1016/j.jaci.2014.03.007 24767877

[8] Karra L Berent-Maoz B Ben-Zimra M Levi-Schaffer F. Are we ready to downregulate mast cells? Curr Opin Immunol (2009) 21:708–14. 10.1016/j.coi.2009.09.010 19837574

[9] Boya P Kroemer G. Lysosomal membrane permeabilization in cell death. Oncogene (2008) 27:6434–51. 10.1038/onc.2008.310 18955971

[10] Turk B Turk V Lysosomes as “suicide bags” in cell death: myth or reality? J Biol Chem (2009) 284:21783–7. 10.1074/jbc.R109.023820 19473965 PMC2755904

[11] Melo FR Waern I Rönnberg E Åbrink M Lee DM Schlenner SM A role for serglycin proteoglycan in mast cell apoptosis induced by a secretory granule-mediated pathway. J Biol Chem (2011) 286:5423–33. 10.1074/jbc.M110.176461 21123167 PMC3037655

[12] Paivandy A Calounova G Zarnegar B Öhrvik H Melo FR Pejler G Mefloquine, an anti-malaria agent, causes reactive oxygen species-dependent cell death in mast cells via a secretory granule-mediated pathway. Pharmacol Res Perspect (2014) 2:e00066 10.1002/prp2.66 25505612 PMC4186446

[13] Spirkoski J Melo FR Grujic M Calounova G Lundequist A Wernersson S Mast cell apoptosis induced by siramesine, a sigma-2 receptor agonist. Biochem Pharmacol (2012) 84:1671–80. 10.1016/j.bcp.2012.09.028 23058984

[14] Ginsburg H. Antimalarial drugs: is the lysosomotropic hypothesis still valid? Parasitol Today (1990) 6:334–7. 10.1016/0169-4758(90)90178-7 15463259

[15] Ostenfeld MS Fehrenbacher N Hoyer-Hansen M Thomsen C Farkas T Jaattela M. Effective tumor cell death by sigma-2 receptor ligand siramesine involves lysosomal leakage and oxidative stress. Cancer Res (2005) 65:8975–83. 10.1158/0008-5472.CAN-05-0269 16204071

[16] Cesen MH Repnik U Turk V Turk B. Siramesine triggers cell death through destabilisation of mitochondria, but not lysosomes. Cell Death Dis (2013) 4:e818. 10.1038/cddis.2013.361 24091661 PMC3824671

[17] Rönnberg E Pejler G. Serglycin: the master of the mast cell. Methods Mol Biol (2012) 836:201–17. 10.1007/978-1-61779-498-8_14 22252637

[18] Metcalfe DD Baram D Mekori YA. Mast cells. Physiol Rev (1997) 77:1033–79. 9354811 10.1152/physrev.1997.77.4.1033

[19] Heading C. Siramesine H Lundbeck. Curr Opin Investig Drugs (2001) 2:266–70. 11816842

[20] Galli SJ Tsai M Wershil BK. The c-kit receptor, stem cell factor, and mast cells. What each is teaching us about the others. Am J Pathol (1993) 142:965–74. 7682764 PMC1886888

[21] Tsai M Shih LS Newlands GF Takeishi T Langley KE Zsebo KM The rat c-kit ligand, stem cell factor, induces the development of connective tissue-type and mucosal mast cells in vivo. Analysis by anatomical distribution, histochemistry, and protease phenotype. J Exp Med (1991) 174:125–31. 10.1084/jem.174.1.125 1711559 PMC2118877

[22] Boesiger J Tsai M Maurer M Yamaguchi M Brown LF Claffey KP Mast cells can secrete vascular permeability factor/vascular endothelial cell growth factor and exhibit enhanced release after immunoglobulin E-dependent upregulation of fc epsilon receptor I expression. J Exp Med (1998) 188:1135–45. 10.1084/jem.188.6.1135 9743532 PMC2212544

[23] Bradding P Feather IH Wilson S Bardin PG Heusser CH Holgate ST Immunolocalization of cytokines in the nasal mucosa of normal and perennial rhinitic subjects. The mast cell as a source of IL-4, IL-5, and IL-6 in human allergic mucosal inflammation. J Immunol (1993) 151:3853–65. 8376806

[24] Burd PR Rogers HW Gordon JR Martin CA Jayaraman S Wilson SD Interleukin 3-dependent and -independent mast cells stimulated with IgE and antigen express multiple cytokines. J Exp Med (1989) 170:245–57. 10.1084/jem.170.1.245 2473161 PMC2189362

[25] Brightling CE Bradding P Symon FA Holgate ST Wardlaw AJ Pavord ID. Mast-cell infiltration of airway smooth muscle in asthma. N Engl J Med (2002) 346:1699–705. 10.1056/NEJMoa012705 12037149

[26] Amin K Janson C Boman G Venge P. The extracellular deposition of mast cell products is increased in hypertrophic airways smooth muscles in allergic asthma but not in nonallergic asthma. Allergy (2005) 60:1241–7. 10.1111/j.1398-9995.2005.00823.x 16134989

[27] Erjefalt JS. Mast cells in human airways: the culprit? Eur Respir Rev (2014) 23:299–307. 10.1183/09059180.00005014 25176966 PMC9487311

[28] Bradding P Arthur G Mast cells in asthma – state of the art. Clin Exp Allergy (2016) 46:194–263. 10.1111/cea.12675 26567481

[29] Dougherty RH Sidhu SS Raman K Solon M Solberg OD Caughey GH Accumulation of intraepithelial mast cells with a unique protease phenotype in T(H)2-high asthma. J Allergy Clin Immunol (2010) 125:1046–53.e8. 10.1016/j.jaci.2010.03.003 20451039 PMC2918406

[30] Andersson CK Bergqvist A Mori M Mauad T Bjermer L Erjefalt JS. Mast cell-associated alveolar inflammation in patients with atopic uncontrolled asthma. J Allergy Clin Immunol (2011) 127:905–12.e1–7. 10.1016/j.jaci.2011.01.022 21388666

[31] Amin K Ludviksdottir D Janson C Nettelbladt O Bjornsson E Roomans GM Inflammation and structural changes in the airways of patients with atopic and nonatopic asthma. BHR Group. Am J Respir Crit Care Med (2000) 162:2295–301. 10.1164/ajrccm.162.6.9912001 11112154

[32] Balzar S Chu HW Strand M Wenzel S. Relationship of small airway chymase-positive mast cells and lung function in severe asthma. Am J Respir Crit Care Med (2005) 171:431–9. 10.1164/rccm.200407-949OC 15563633

[33] Andersson CK Andersson-Sjoland A Mori M Hallgren O Pardo A Eriksson L Activated MCTC mast cells infiltrate diseased lung areas in cystic fibrosis and idiopathic pulmonary fibrosis. Respir Res (2011) 12:139. 10.1186/1465-9921-12-139 22014187 PMC3209449

[34] Andersson CK Mori M Bjermer L Lofdahl CG Erjefalt JS. Alterations in lung mast cell populations in patients with chronic obstructive pulmonary disease. Am J Respir Crit Care Med (2010) 181:206–17. 10.1164/rccm.200906-0932OC 19926870

[35] Yu M Tsai M Tam SY Jones C Zehnder J Galli SJ. Mast cells can promote the development of multiple features of chronic asthma in mice. J Clin Invest (2006) 116:1633–41. 10.1172/JCI25702 16710480 PMC1462940

[36] Reber LL Daubeuf F Pejler G Abrink M Frossard N. Mast cells contribute to bleomycin-induced lung inflammation and injury in mice through a chymase/mast cell protease 4-dependent mechanism. J Immunol (2014) 192:1847–54. 10.4049/jimmunol.1300875 24453258

[37] Beckett EL Stevens RL Jarnicki AG Kim RY Hanish I Hansbro NG A new short-term mouse model of chronic obstructive pulmonary disease identifies a role for mast cell tryptase in pathogenesis. J Allergy Clin Immunol (2013) 131:752–62. 10.1016/j.jaci.2012.11.053 23380220 PMC4060894

[38] Hansbro PM Hamilton MJ Fricker M Gellatly SL Jarnicki AG Zheng D Importance of mast cell Prss31/transmembrane tryptase/tryptase-gamma in lung function and experimental chronic obstructive pulmonary disease and colitis. J Biol Chem (2014) 289:18214–27. 10.1074/jbc.M114.548594 24821729 PMC4140286

[39] Asai K Kanazawa H Kamoi H Shiraishi S Hirata K Yoshikawa J. Increased levels of vascular endothelial growth factor in induced sputum in asthmatic patients. Clin Exp Allergy (2003) 33:595–9. 10.1046/j.1365-2222.2003.01576.x 12752587

[40] Kanazawa H Nomura S Yoshikawa J. Role of microvascular permeability on physiologic differences in asthma and eosinophilic bronchitis. Am J Respir Crit Care Med (2004) 169:1125–30. 10.1164/rccm.200401-123OC 15044203

[41] Kanazawa H Hirata K Yoshikawa J. Involvement of vascular endothelial growth factor in exercise induced bronchoconstriction in asthmatic patients. Thorax (2002) 57:885–8. 10.1136/thorax.57.10.885 12324676 PMC1746203

[42] Detoraki A Staiano RI Granata F Giannattasio G Prevete N de Paulis A Vascular endothelial growth factors synthesized by human lung mast cells exert angiogenic effects. J Allergy Clin Immunol (2009) 123:1142–9, 1149.e1–5. 10.1016/j.jaci.2009.01.044 19275959

[43] Yokoyama A Kohno N Fujino S Hamada H Inoue Y Fujioka S Circulating interleukin-6 levels in patients with bronchial asthma. Am J Respir Crit Care Med (1995) 151:1354–8. 10.1164/ajrccm.151.5.7735584 7735584

[44] Morjaria JB Babu KS Vijayanand P Chauhan AJ Davies DE Holgate ST Sputum IL-6 concentrations in severe asthma and its relationship with FEV1. Thorax (2011) 66:537 10.1136/thx.2010.136523 20880874

[45] Tillie-Leblond I Pugin J Marquette CH Lamblin C Saulnier F Brichet A Balance between proinflammatory cytokines and their inhibitors in bronchial lavage from patients with status asthmaticus. Am J Respir Crit Care Med (1999) 159:487–94. 10.1164/ajrccm.159.2.9805115 9927362

[46] Neveu WA Allard JL Raymond DM Bourassa LM Burns SM Bunn JY Elevation of IL-6 in the allergic asthmatic airway is independent of inflammation but associates with loss of central airway function. Respir Res (2010) 11:28. 10.1186/1465-9921-11-28 20205953 PMC2842243

